# Impact of milk consumption patterns on cow's milk sensitization and allergy in at-risk children^☆^

**DOI:** 10.1016/j.waojou.2026.101376

**Published:** 2026-04-07

**Authors:** Liat Azani, Mali Salmon-Divon, Gabi Gerlitz, Michael Brandwein

**Affiliations:** a Department of Molecular Biology, Ariel University, Ariel 40700, Israel; b Adelson School of Medicine, Ariel University, Ariel 40700, Israel

**Keywords:** Cow's milk, Milk allergy, Formula, Milk sensitization, Breastfeeding

## Abstract

**Background:**

Cow's milk allergy is the most common food allergy in infants. While early peanut consumption can reduce peanut allergy risk, the effects of cow's milk formula consumption patterns on milk sensitization and allergy are not well understood.

**Objective:**

To examine the association between early cow's milk formula consumption patterns and cow's milk sensitization and allergy in high-risk infants.

**Methods:**

We conducted a secondary cross-sectional analysis of 416 infants (3–15 months) from the CoFAR study, categorized by milk consumption patterns: hospital consistent, hospital inconsistent, home only, and non-consumers. Group differences were assessed using Kruskal–Wallis and post-hoc tests. Univariate and multivariate logistic regression models estimated associations with cow's milk sensitization and allergy.

**Results:**

Hospital consistent infants had smaller SPT wheal sizes and less sensitization than hospital inconsistent and non-consumers. Early inconsistent consumption was associated with higher odds of sensitization (OR = 2.45; 95% CI: 1.24–5.05), while non-consumption was associated with lower allergy odds (OR = 0.39; 95% CI: 0.21–0.69). As clinical CMA diagnosis requires prior ingestion with reaction, lower allergy rates among non-consumers may reflect limited exposure opportunities rather than a protective effect. Home-only consumption did not significantly affect risk.

**Conclusions:**

Early cow's milk formula consumption patterns were associated with differences in cow's milk sensitization and allergy outcomes in high-risk infants. Consistent exposure was associated with lower sensitization, whereas non-consumption was associated with lower allergy prevalence, highlighting the potential role of early feeding practices in allergy prevention.

## Introduction

Pediatric food allergies are increasingly prevalent,^1^ with rates rising steadily over the past few decades. While the underlying causes remain under investigation, cow's milk allergy (CMA) is the most common food allergy in infants and young children, affecting approximately 2–3% of children under 3 years old.^1^ The Learning Early About Peanut Allergy (LEAP) study, published in 2015, demonstrated that early and regular peanut exposure significantly reduced peanut allergy risk in high-risk infants,^2^ This landmark trial, which included over 600 infants aged 4–11 months, showed that early introduction and consistent consumption of peanuts lowered the risk of peanut allergy by up to 81% in high-risk infants.^2^ These findings reshaped allergy prevention guidelines and spurred investigations into similar strategies for other allergens, including milk.^3^, ^4^, ^5^ However, important differences exist between peanut allergy and CMA, including natural history and typical timing of exposure, and therefore LEAP-like comparisons should be interpreted cautiously. Unlike most allergens, which are typically introduced during complementary feeding, cow's milk protein is often consumed earlier through cow's milk formula (CMF). Moreover, the present study examines a cohort already enriched for food allergy and atopic dermatitis, which has implications for generalizability and interpretation. However, research on its impact on cow's milk sensitization (CMS) and CMA development has been limited by the wide variability in the timing of formula introduction.^6^ We hypothesize that different patterns of milk consumption during infancy may influence the development of milk allergies later in life. Understanding the mechanisms of early exposure to cow's milk in a high-risk population may yield important insights into strategies for primary prevention of CMA.

## Methods

**Fig. 1:**
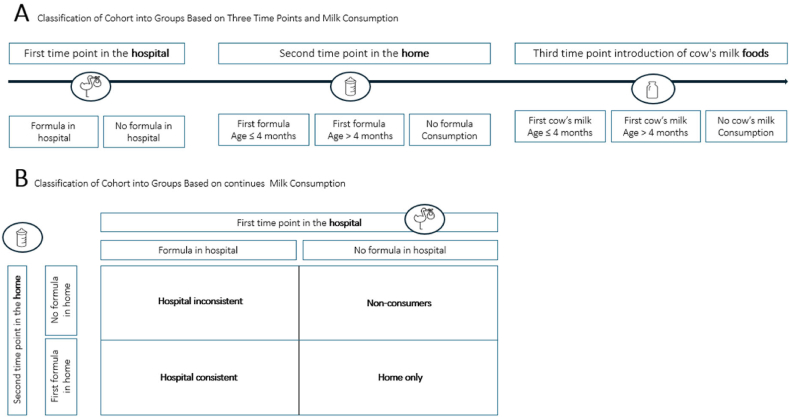
**Study Design: Classification of formula exposure groups**. Panel A presents the three time points used to characterize milk consumption patterns, and Panel B shows the simplified grouping used in this study, based on continuous formula exposure during routine birth hospitalization period and at home.

We conducted a secondary analysis of data from the CoFAR study,^7^ a multi-center, cross-sectional, observational investigation aimed at examining the natural progression of peanut allergy in high-risk infants aged 3–15 months, who also had a pre-existing allergy to egg and/or milk, or moderate to severe atopic dermatitis. Given the cross-sectional design, temporality between feeding patterns and outcomes cannot be inferred. Clinical outcome measures used were baseline CMS, defined as a skin prick test (SPT) wheal size ≥3, and baseline CMA defined according to CoFAR diagnostic categories as follows: infants were classified as allergic if they met criteria for either confirmed IgE-mediated allergy (positive oral food challenge and sensitization, or convincing reaction with highly predictive IgE levels), or convincing but unconfirmed allergy (clinical history consistent with IgE-mediated reaction and sensitization, but without oral food challenge and below predictive IgE thresholds). All other participants were classified as non-allergic which was defined according to the study protocol.^7^^,^^8^ We divided the cohort into 4 groups based on parent-reported milk consumption patterns: “hospital consistent” (formula consumption in the hospital and at home, n = 142, 34.1%, Supplementary Table 1), “hospital inconsistent” (formula consumption in the hospital and not at home, n = 71, 17%), “home only” (formula consumption at home, n = 107, 25.7%), and “non-consumers” (n = 96, 23%). Milk consumption was assessed at 2 distinct time points: during routine birth hospitalization period, when infants were assessed during the immediate postnatal stay, meaning within the first days of life, and again in the home setting after discharge. Fig. 1 illustrates the classification of formula exposure groups. Panel A presents the 3 time points used to characterize milk consumption patterns, while Panel B shows the simplified grouping used in this study, based on continuous formula exposure during routine birth hospitalization period and at home. Demographic data, descriptive statistics and univariate analysis were available for 416 participants, and have been described previously.^8^

Differences between groups were initially assessed using the Kruskal–Wallis test, followed by post-hoc tests such as Dunn's test or FDR corrected. A univariate analysis was conducted to identify pivotal variables and confounders and to improve the robustness of multivariate models. Variables with significance thresholds of 0.2 were included in a subsequent multivariate analyses, consistent with recommended model-building approaches that use a more liberal screening threshold to avoid excluding potentially important confounders. Adjusted odds ratios (aORs) and their corresponding 95% confidence intervals (CIs) were computed to gauge the strength and significance of the associations concerning milk sensitization.

## Results

**Fig. 2:**
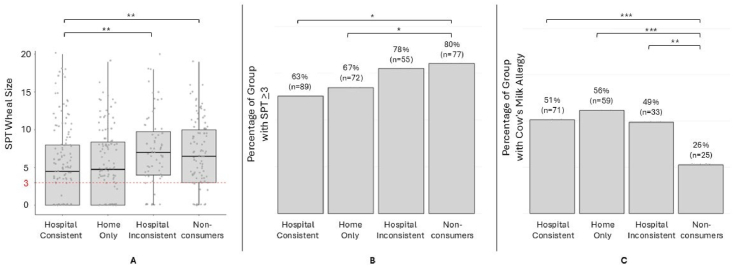
**Diagram A illustrates the classification of the cohort based on 3 time points: formula consumption during routine birth hospitalization period, age at first formula consumption at home (≤4 months or >4 months), and age at first cow's milk food introduction. Diagram B presents the simplified classification used in this study, based on continuity of formula exposure across two-time points hospital and home resulting in 4 distinct groups: hospital consistent, hospital inconsistent, home only, and non-consumers.** **SPT wheal size, sensitization and allergy to milk by study groups:** (A) The horizontal red dashed line represents the threshold for cow's milk sensitivity. Individual data points are shown as grey dots. A significant pairwise comparison (Dunn's) is marked between the hospital consistent group and the non-consumers group. (B) Cow's milk sensitization (SPT Wheal Size ≥3) occurred significantly less in the hospital consistent group when compared to the hospital inconsistent groups (p < 0.05) and the non-consumer group (p < 0.05, pairwise Chi-Square). (C) Cow's milk allergy occurred significantly less in the non-consumer group when compared to the hospital inconsistent (p < 0.01), hospital consistent (p < 0.001) and the home only group (p < 0.001, pairwise Chi-Square)

SPT wheal sizes were significantly different between groups (p < 0.05, Kruskal-Wallis). They were smaller in the hospital consistent group when compared to the hospital inconsistent groups (p < 0.05) and the non-consumer group (p < 0.05, Dunn's Test, Fig. 2a). Sensitization occurred significantly less in the hospital consistent group when compared to the hospital inconsistent groups (FDR-corrected p < 0.05, pairwise Chi-Square, Fig. 2b) and the non-consumer group (p < 0.05). No significant differences were observed between the home-only group and any of the other groups for SPT wheal size or CMS. CMA occurred significantly less in the non-consumer group when compared to the hospital inconsistent (FDR-corrected p < 0.01, pairwise Chi-Square, Fig. 2c), hospital consistent (p < 0.001) and the home only group (p < 0.001). Importantly, the diagnostic criteria for confirmed or convincing CMA necessitated either previous ingestion with reaction or flare or an indicated oral food challenge. The former was not conducted on all study participants.

Compared to the hospital consistent group, the hospital inconsistent group (OR = 2.53; 95% CI: 1.29–5.23, p < 0.01) and non-consumers (OR = 2.43; 95% CI: 1.34–4.55, p < 0.01) were associated with higher odds of CMS in a univariate model, and the non-consumers were associated with a lower odds of CMA in a separate univariate model (OR = 0.34; 95% CI: 0.19–0.60, p < 0.001, Supplementary Table 2 and Supplementary Table 3). A multivariate logistic regression model, which included variables with a significance threshold of 0.2 in the univariate analysis, identified significant risk factors for CMS (Table 1). Early inconsistent milk consumption (hospital inconsistent) was significantly associated with a higher likelihood of CMS (OR = 2.45, 95% CI: 1.24–5.05, p = 0.01) compared to early consistent consumption (hospital consistent). Milk consumption at home but not in the hospital (home only) was not associated with risk for CMS (OR = 0.96, 95% CI: 0.54–1.69, p = 0.88). No CMF consumption showed a marginally significant association with CMS (OR = 1.71, 95% CI: 0.89–3.36, p = 0.10). In a separate multivariate model for CMA, non-consumers were significantly less likely to meet criteria for CMA (OR = 0.39, 95% CI: 0.21–0.69, p < 0.001) than the hospital consistent group and age at enrollment had a significant positive association with CMA (OR = 1.08, 95% CI: 1.00–1.18, p < 0.05).

**Table 1:** Multivariable logistic regression analysis models for milk sensitization and milk allergy adjusted for milk continuation, and demographic characteristics.

Characteristic		Milk Sensitization			Milk Allergy		
		Adjusted OR	95% CI	p-value	Adjusted OR	95% CI	p-value
Milk consumption	Hospital consistent	ref			ref		
	Hospital inconsistent	2.45	1.24–5.05	**0.01**	1.02	0.56–1.86	0.93
	Home only	0.96	0.54–1.69	0.88	1.27	0.76–2.14	0.35
	Non-consumers	1.71	0.89–3.36	0.1	0.39	0.21–0.69	**>0.001**
Breastfeeding	Never	ref					
	Yes, currently	2.08	0.98–4.39	0.054			
	Yes, but no longer	1.72	0.84–3.49	0.12			
Maternal Smoking	No	ref					
	Yes	0.43	0.22–0.84	**0.01**			
Maternal atopic dermatitis	No	ref					
	Yes	1.57	0.89–2.82	0.12			
	Unknown	1.71	1.02–2.91	**0.04**			
Siblings	No	ref			ref		
	Yes	1.34	0.84–2.13	0.2	0.74	0.47–1.14	0.17
Ethnicity	Other	ref					
	White or Caucasian	1.33	0.81–2.17	0.23			
Dog in Household	No				ref		
	Yes				1.41	0.91–2.18	0.11
Birthweight					0.88	0.60–1.28	0.52
Age at enrollment					1.08	1.00–1.18	**0.04**

Bolded values indicate statistical significance at the *p < .05* level.

OR = Odds Ratio, CI = Confidence Interval. Multivariate models included variables with a significance threshold of 0.2 in the univariate analysis

## Discussion

Our findings suggest that early and consistent milk consumption was associated with a smaller SPT wheal size later on. Non-consumers of milk formula were less likely to be diagnosed with CMA by study enrollment, potentially due to the lack of consumption of cow's milk by study enrollment. These results align with previous evidence from the LEAP trial, which demonstrated the potential of early and sustained exposure to reduce allergy risk for peanuts, and support the concept that similar mechanisms may apply to other allergens although important differences between peanut allergies and CMA limit the direct comparability of these findings. This early window is clinically meaningful, as evidence from randomized trials suggests that exposure to cow's milk formula during the first days of life may influence the risk of later sensitization. For example, the ABC trial demonstrated that avoiding cow's milk formula supplementation during at least the first 3 days of life significantly reduced the risk of cow's milk sensitization and food allergy by age 2.^9^ Nevertheless, the interpretation of our results should be made with caution due to limitations including sample size, the reliance on parental report for milk consumption patterns, which were categorized broadly and may encompass heterogeneous exposures in terms of quantity, frequency, and duration, introducing potential exposure misclassification and recall bias and the high-risk nature of our cohort. Importantly, the cross-sectional design precludes establishing temporality, and therefore the associations observed cannot be interpreted as causal. While the findings may not be directly generalizable to the general population, they are highly clinically relevant for high-risk groups and thus represent an important contribution to existing knowledge. Further research is needed to confirm these findings in larger cohorts, the general population, and to explore underlying mechanisms that may help clarify observed associations.

## Conclusions

Our findings suggest that patterns of cow's milk consumption in infancy were associated with differences in cow's milk allergy classification at enrollment. While preliminary, these results highlight the potential clinical importance of early feeding practices, particularly for high-risk groups. Larger, prospective studies are needed to validate these observations and to guide evidence-based strategies for allergy prevention.

## Funding sources

This study did not receive any funding.

## References

[1] Giannetti A., Toschi Vespasiani G., Ricci G., Miniaci A., di Palmo E., Pession A. (2021). Cow's milk protein allergy as a model of food allergies. Nutrients.

[2] Du Toit G., Roberts G., Sayre P.H. (2015). Randomized trial of peanut consumption in infants at risk for peanut allergy. N Engl J Med.

[3] Sampath V., Abrams E.M., Adlou B. (2021). Food allergy across the globe. J Allergy Clin Immunol.

[4] Sakihara T., Otsuji K., Arakaki Y., Hamada K., Sugiura S., Ito K. (2021). Randomized trial of early infant formula introduction to prevent cow's milk allergy. J Allergy Clin Immunol.

[5] Lachover-Roth I., Cohen-Engler A., Furman Y. (2023). Early, continuing exposure to cow's milk formula and cow's milk allergy: the COMEET study, a single center, prospective interventional study. Ann Allergy Asthma Immunol.

[6] Brandwein M., Enten Vissoker R., Jackson H. (2024). Redefining the role of nutrition in infant food allergy prevention: a narrative review. Nutrients.

[7] Sampson H.A., Berin M.C., Plaut M. (2019). The consortium for food allergy research (CoFAR): the first generation. J Allergy Clin Immunol.

[8] Azani L., Landau T., Brandwein M., Salmon-Divon M. (2025). The effect of infant cow's milk protein consumption on subsequent IgE-mediated cow's milk allergic outcomes in a high-risk pediatric population. Clinical Nutrition.

[9] Kido H., Sakurai D., Imai T. (2020). Primary prevention of cow's milk sensitization and food allergy by avoiding supplementation with cow's milk formula at birth: a randomized clinical trial. JAMA Pediatr.

