# Supplementary material for: Impact of milk consumption patterns on cow's milk sensitization and allergy in at-risk children
Source: World Allergy Organ J. 2026 Apr 7;19(4):101376. doi: 10.1016/j.waojou.2026.101376 (PMC13090969; doi:10.1016/j.waojou.2026.101376)
Supplement: Multimedia component 1 [file mmc1.docx]

**Supplementary** **Information**

*Supplementary Table 1*: Characteristics of Study Population

| **CMS** | **Total, N=416** | **SPT-, N=123** | **SPT+, N=293** | **p-value** |
| --- | --- | --- | --- | --- |
| Hospital Consistent | 142 (100) | 53 (37.3) | 89 (62.7) | 0.013 |
| Hospital Inconsistent | 71 (100) | 16 (22.5) | 55 (77.5) |  |
| Home Only | 107 (100) | 35 (32.7) | 72 (67.3) |  |
| Non-Consumers | 96 (100) | 19 (19.8) | 77 (80.2) |  |
| **CMA** | **Total, N=408** | **Allergy-, N=220** | **Allergy+, N=188** | **p-value** |
| Hospital Consistent | 140 (100) | 69 (49.3) | 71 (50.7) | >0.001 |
| Hospital Inconsistent | 67 (100) | 34 (50.7) | 33 (49.3) |  |
| Home Only | 106 (100) | 47 (44.3) | 59 (55.7) |  |
| Non-Consumers | 95 (100) | 70 (73.7) | 25 (26.3) |  |

Abbreviations: SPT, skin prick test

Chi-squared test was used to assess significance between clinical outcome measures and cow's milk consumption groups.

*Supplementary Table* 2: Post-hoc analysis of pairwise group differences in cow's milk sensitivity and allergy outcomes

|  | | Sensitivity | | Allergy | |
| --- | --- | --- | --- | --- | --- |
| Pairwise Groups | | p-value | Adjusted p-value | p-value | Adjusted p-value |
| Home only | Non-consumers | 0.0563 | 0.1126 | 0.0000 | **0.0002** |
| Home only | Hospital consistent | 0.4193 | 0.5031 | 0.5190 | 0.6230 |
| Home only | Hospital inconsistent | 0.0803 | 0.1204 | 0.4370 | 0.6230 |
| Non-consumers | Hospital consistent | 0.0039 | **0.0235** | 0.0002 | **0.0007** |
| Non-consumers | Hospital inconsistent | 1.0000 | 1.0000 | 0.0045 | **0.0089** |
| Hospital consistent | Hospital inconsistent | 0.0104 | **0.0311** | 0.8830 | 0.8826 |

Pairwise Chi-squared test was used to assess significance between clinical outcome measures and paired cow's milk consumption groups. FDR adjusted p-values to control for Type I error.

*Supplementary Table* 3: Univariable logistic regression analysis model for cow's milk sensitivity and allergy and demographic characteristics

|  | | Sensitivity | | Allergy | |
| --- | --- | --- | --- | --- | --- |
| Characteristic | | Adjusted OR (95%CI) | p-value | Adjusted OR (95%CI) | p-value |
| Milk formula | No |  |  |  |  |
|  | Yes | 0.75 (0.49-1.15) | 0.196 | 1.40 (0.95-2.08) | **0.07** |
| CMF consumption at home | Early |  |  |  |  |
|  | Late | 1.43 (0.72-2.98) | 0.31 | 1.84 (0.94-3.74) | **0.07** |
|  | Nonformula | 2.19 (1.38- 3.53) | **<0.001** | 0.55 (0.36-0.84) | **<0.001** |
| First cow’s milk consumption | Early |  |  |  |  |
|  | Late | 1.80 (0.88-3.72) | 0.106 | 1.08 (0.52-2.25) | 0.83 |
|  | Nonformula | 5.77 (3.06-11.08) | **<0.001** | 1.44 (0.77-2.72) | 0.25 |
| Milk Consumption Groups | Hospital Consistent |  |  |  |  |
|  | Hospital Inconsistent | **2.53 (1.29-5.23)** | **0.008** | 0.94 (0.52-1.69) | 0.844 |
|  | Home Only | 1.29 (0.76-2.20) | 0.348 | 1.21 (0.73-2.02) | 0.441 |
|  | Non-consumers | **2.43 (1.34-4.55)** | **0.004** | 0.34 (0.19-0.60) | **<0.001** |
| Breastfeeding | Never |  |  |  |  |
|  | Yes currently | 2.66 (1.41-5.01) | **0.002** | 0.68 (0.37-1.25) | 0.21 |
|  | Yes, but no longer | 1.77 (0.97-3.21) | **0.06** | 0.72 (0.40-1.31) | 0.29 |
| Gender | Male |  |  |  |  |
|  | Female (1) | 1.11 (0.71-1.76) | 0.63 | 1.32 (0.87-2.00) | 0.18 |
| Full term pregnancy (>=37 Weeks) | No |  |  |  |  |
|  | Yes | 1.36 (0.56-3.12) | 0.46 | 0.92 (0.40-2.09) | 0.84 |
| Mode of delivery | No caesarian |  |  |  |  |
|  | Caesarian | 0.94 (0.60-1.48) | 0.80 | 0.98 (0.64-1.49) | 0.93 |
| Mother smoked | No |  |  |  |  |
|  | Yes | 0.39 (0.21-0.65) | **0.001** | 0.93 (0.52-1.64) | 0.81 |
| Maternal atopic dermatitis | No |  |  |  |  |
|  | Yes | 1.45 (0.84-2.54) | 0.18 | 0.69 (0.41-1.14) | 0.15 |
|  | Unknown | 1.43 (0.88-2.35) | 0.14 | 1.02 (0.65-1.59) | 0.92 |
| Maternal food allergy | No |  |  |  |  |
|  | Yes | 1.00 (0.57-1.77) | 0.99 | 1.10 (0.64-1.88) | 0.71 |
|  | Unknown | 1.29 (0.79-2.10) | 0.30 | 1.27 (0.82-1.97) | 0.27 |
| Maternal asthma | No |  |  |  |  |
|  | Yes | 0.75 (0.44-1.28) | 0.29 | 0.97 (0.58-1.62) | 0.92 |
|  | Unknown | 1.18 (0.72-1.95) | 0.50 | 1.17 (0.75-1.83) | 0.48 |
| Paternal atopic dermatitis | No |  |  |  |  |
|  | Yes | 0.98 (0.55-1.75) | 0.95 | 1.08 (0.63-1.86) | 0.76 |
|  | Unknown | 1.24 (0.77-2.00) | 0.36 | 1.31 (0.85-2.03) | 0.22 |
| Paternal food allergy | No |  |  |  |  |
|  | Yes | 1.08 (0.58-2.04) | 0.80 | 0.97(0.54-1.74) | 0.93 |
|  | Unknown | 1.19 (0.75-1.89) | 0.45 | 1.22 (0.80-1.86) | 0.35 |
| Paternal asthma | No |  |  |  |  |
|  | Yes | 0.92 (0.53-1.62) | 0.79 | 1.00 (0.59-1.70) | 0.97 |
|  | Unknown | 1.18 (0.73-1.92) | 0.48 | 1.27 (0.82-1.98) | 0.27 |
| Siblings | No |  |  |  |  |
|  | Yes | 1.38 (0.88-2.13) | 0.14 | 0.70 (0.46-1.06) | **0.09** |
| Ethnicity | Other |  |  |  |  |
|  | White or Caucasian | 1.53 (0.96-2.41) | **0.06** | 1.18 (0.77-1.84) | 0.43 |
| Cat in household | No |  |  |  |  |
|  | Yes | 0.69 (0.37-1.30) | 0.24 | 0.78 (0.42-1.42) | 0.43 |
| Dog in household | No |  |  |  |  |
|  | Yes | 0.98 (0.63-1.54) | 0.94 | 1.44 (0.96-2.18) | **0.07** |
| Birthweight | Mean (SD) | 1.19 (0.81-1.74) | 0.36 | 0.77 (0.53-1.10) | 0.16 |
| Age at enrollment | Mean (SD) | 0.98 (0.90-1.06) | 0.68 | 1.10(1.02-1.200 | **0.01** |
